# Biomedical Waste Management and Its Importance: A Systematic Review

**DOI:** 10.7759/cureus.34589

**Published:** 2023-02-03

**Authors:** Himani S Bansod, Prasad Deshmukh

**Affiliations:** 1 Community Medicine, Jawaharlal Nehru Medical College, Datta Meghe Institute of Higher Education and Research, Wardha, IND; 2 Head and Neck Surgery, Jawaharlal Nehru Medical College, Datta Meghe Institute of Higher Education and Research, Wardha, IND

**Keywords:** colour coding, transportation, segregation, healthcare workers, knowledge, biomedical waste

## Abstract

The waste generated in various hospitals and healthcare facilities, including the waste of industries, can be grouped under biomedical waste (BMW). The constituents of this type of waste are various infectious and hazardous materials. This waste is then identified, segregated, and treated scientifically. There is an inevitable need for healthcare professionals to have adequate knowledge and a proper attitude towards BMW and its management. BMW generated can either be solid or liquid waste comprising infectious or potentially infectious materials, such as medical, research, or laboratory waste. There is a high possibility that inappropriate management of BMW can cause infections to healthcare workers, the patients visiting the facilities, and the surrounding environment and community. BMW can also be classified into general, pathological, radioactive, chemical, infectious, sharps, pharmaceuticals, or pressurized wastes. India has well-established rules for the proper handling and management of BMW. Biomedical Waste Management Rules, 2016 (BMWM Rules, 2016) specify that every healthcare facility shall take all necessary steps to ensure that BMW is handled without any adverse effect on human and environmental health. This document contains six schedules, including the category of BMW, the color coding and type of containers, and labels for BMW containers or bags, which should be non-washable and visible. A label for the transportation of BMW containers, the standard for treatment and disposal, and the schedule for waste treatment facilities such as incinerators and autoclaves are included in the schedule. The new rules established in India are meant to improve the segregation, transportation, disposal methods, and treatment of BMW. This proper management is intended to decrease environmental pollution because, if not managed properly, BMW can cause air, water, and land pollution. Collective teamwork with committed government support in finance and infrastructure development is a very important requirement for the effective disposal of BMW. Devoted healthcare workers and facilities are also significant. Further, the proper and continuous monitoring of BMW is a vital necessity. Therefore, developing environmentally friendly methods and the right plan and protocols for the disposal of BMW is very important to achieve a goal of a green and clean environment. The aim of this review article is to provide systematic evidence-based information along with a comprehensive study of BMW in an organized manner.

## Introduction and background

The amount of daily biomedical waste (BMW) produced in India is enormous [1]. People from all segments of society, regardless of age, sex, ethnicity, or religion, visit hospitals, which results in the production of BMW, which is becoming increasingly copious and heterogeneous [2]. BMW produced in India is about 1.5-2 kg/bed/day [3]. BMW include anatomical waste, sharps, laboratory waste, and others and, if not carefully segregated, can be fatal. Additionally, inappropriate segregation of dirty plastic, a cytotoxic and recyclable material, might harm our ecosystem [4]. Earlier, BMW was not considered a threat to humans and the environment. In the 1980s and 1990s, fears about contact with infectious microorganisms such as human immunodeficiency virus (HIV) and hepatitis B virus (HBV) prompted people to consider the potential risks of BMW [5]. BMW is hazardous in nature as it consists of potential viruses or other disease-causing microbial particles; it may be present in human samples, blood bags, needles, cotton swabs, dressing material, beddings, and others. Therefore, the mismanagement of BMW is a community health problem. The general public must also take specific actions to mitigate the rising environmental degradation brought on by negligent BMW management. On July 20, 1998, BMW (Management and Handling) Rules were framed. On March 28, 2016, under the Environment (Protection) Act, 1986, the Ministry of Environment and Forest (MoEF) implemented the new BMW Rules (2016) and replaced the earlier one (1988). BMW produced goes through a new protocol or approach that helps in its appropriate management in terms of its characterization, quantification, segregation, storage, transport, and treatment.

According to Chapter 2 of the Medical Waste Management and Processing Rules, 2016, “The BMW could not be mixed with other wastes at any stage while producing inside hospitals, while collecting from hospitals, while transporting, and should be processed separately based on classification.” The COVID-19 pandemic has now transformed healthy societies worldwide into diseased ones, resulting in a very high number of deaths. It also created one significant problem: improper handling of the medical waste produced in the testing and treatment of the disease [6]. In India, BMW generated due to COVID-19 contributed to about 126 tonnes per day out of the 710 tonnes of waste produced daily [7]. 

The basic principle of the management of BMW is Reduce, Reuse, and Recycle-the 3Rs. Out of the total amount of BMW generated, 85% is general (non-hazardous) waste, and the remaining 15% is hazardous. As BMW contains sharps and syringes, the pathogens can enter the human body through cuts, abrasions, puncture wounds, and other ways. There might also be chances of ingestion and inhalation of BMW, which can lead to infections. Some examples of infections are Salmonella, Shigella, Mycobacterium tuberculosis, Streptococcus pneumonia, acquired immunodeficiency syndrome (AIDS), hepatitis A, B, and C, and helminthic infections [8]. This systematic review is conducted to obtain essential, up-to-date information on BMW for the practical application of its management. The highlight of the management of BMW is that the “success of BMW management depends on segregation at the point of generation” [9].

## Review

Method

The findings have been reported following the principles and criteria of the Preferred Reporting Items for Systematic Review and Meta-Analysis (PRISMA). The systematic review has been conducted according to these standards and principles.

Search Sources/Search Strategy

We used the MeSH strategy to obtain articles from PubMed and ResearchGate employing the following terms: (“Biomedical/waste” [Majr] OR “Biomedical Waste/source” [Majr] OR “Biomedical Waste/hazards” [Majr] OR “Biomedical Waste/segregation” [Majr] OR “Biomedical Waste/rules” [Majr] OR “Biomedical Waste/laws” [Majr] OR “Biomedical Waste/environment” [Majr]). Specifically, for management-related studies, the search terms (“Management/steps” [Majr] OR “Management/handling” [Majr] OR “Management/coding” [Majr] OR “Color coding/segregation” [Majr] OR “Treatment/method” [Majr] OR “Autoclaving/waste” [Majr] OR “Incineration/waste” [Majr]) were used. We obtained the most pertinent research papers and used them in different arrangements using the Boolean operators “AND” and “OR.”

Inclusion and exclusion criteria

We focused on papers written in the English language, published within the last decade, relevant to the central questions of this review article, and that are systematic reviews such as randomized clinical trials and observational studies. We, however, excluded papers published in languages other than English, irrelevant to the questions, and related to topics other than BMW.

Results

Search outcomes

**Figure 1:**
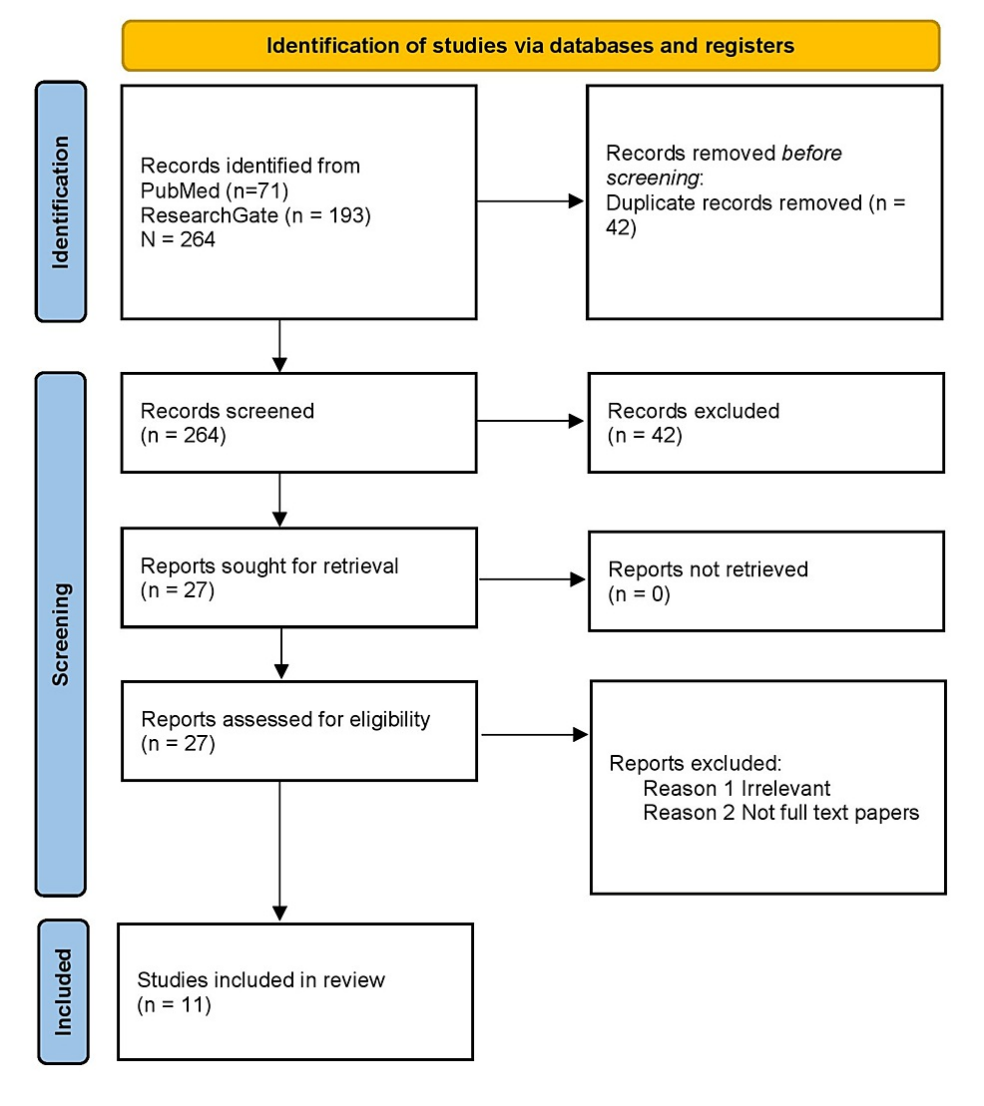
Figure 1: PRISMA 2020 flow chart for systematic review. PRISMA: Preferred Reporting Items for Systematic Review and Meta-analysis, PMC: PubMed Central

After the initial screening, we narrowed the search results down to 264 papers. A total of 42 duplicate papers were removed. Subsequently, publications were refined by the title/abstract, and we eliminated a few studies due to the lack of full text and/or related articles. Finally, after assessing 27 items for eligibility, we included 11 papers in our review. Figure 1 is the flow chart for article selection formulated on PRISMA.

Need for BMW management in hospitals

BMW threatens the health of medical staff, hospital-visiting patients, and people in the nearby community. Improper disposal leads to severe hospital-acquired diseases along with an increased risk of air and water pollution. Due to open-space waste disposal practices, animals and scavengers might get infected, leading to the scattering of waste and the spreading of infections. In countering such activities, four major principle functions of BMW management are applicable: the placement of bins at the source of generation of BMW, segregation of BMW, removal or mutilation of the recyclable waste, and disinfection of the waste [10]. BMW management methods aim predominantly to avoid the generation of waste and, if generated, then recover as much as possible [11].

BMW management rules in India

On March 28, 2016, under the Environment (Protection) Act, 1986, the MoEF notified the new BMW Rules, 2016 and replaced the earlier Rules (1988). BMW produced goes through a new protocol or approach which helps in the appropriate management of waste, i.e., its characterization, quantification, segregation, storage, transport, and treatment, all of which aim to decrease environmental pollution [12]. Problems with the improper management of BMW also shed light on the scavengers who, for recycling, segregate the potentially hazardous BMW without using gloves or masks. Strict rules have been implemented to ensure that there is no stealing of recyclable materials or spillage by some humans or animals and that it is transported to the common BMW treatment facility [10]. The first solution to stop the spread of hazardous and toxic waste was incineration. Incineration is required in all hospitals and healthcare facilities that produce BMW. However, due to the absence of services that provide certified incinerators in a few countries, BMW has to be sent to landfills, which leads to land contamination and harms the environment [13]. Incinerators used for disposal might also lead to environmental pollution. Numerous toxins are formed during incineration, which are the products of incomplete combustion. Thus, some new standards have been issued to resolve this problem and safeguard the environment and public health [14].

Steps in the management of BMW

BMW management needs to be organized, as even a single mistake can cause harm to the people in charge. There are six steps in the management of BMW [15]: surveying the waste produced; segregating, collecting, and categorizing the waste; storing, transporting, and treating the waste. Segregation is the separation of different types of waste generated, which helps reduce the risks resulting from the improper management of BMW. When the waste is simply disposed of, there is an increased risk of the mixture of waste such as sharps with general waste. These sharps can be infectious to the handler of the waste. Further, if not segregated properly, there is a huge chance of syringes and needles disposed of in the hospitals being reused. Segregation prevents this and helps in achieving the goal of recycling the plastic and metal waste generated [16]. According to Schedule 2, waste must be segregated into containers at the source of its generation, and according to Schedule 3, the container used must be labeled. The schedules of BMW (Management and Handling) Rules, 1998, which were initially ten in number, have now been reduced to four [17]. The collection of BMW involves the use of different colors of bins for waste disposal. The color is an important indicator for the segregation and identification of different categories of waste into suitable-colored containers. They must be labeled properly based on the place they have been generated, such as hospital wards, rooms, and operation theatres. It is also very important to remember that the waste must be stored for less than 8-10 hours in hospitals with around 250 beds and 24 hours in nursing homes. The storage bag or area must be marked with a sign [16]. 

Figure 1 shows the biohazard signs that symbolize the nature of waste to the general public.

**Figure 2:**
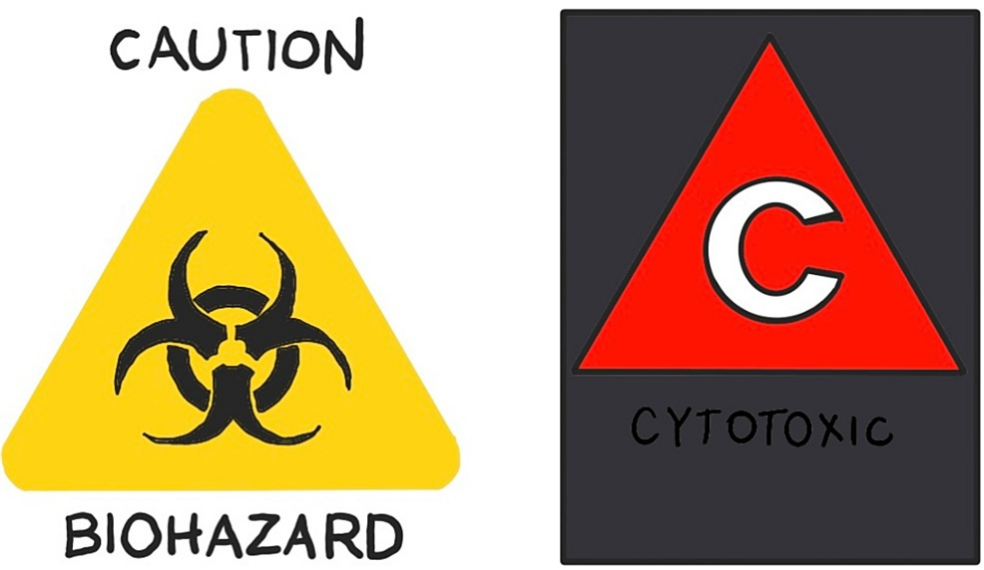
Biohazard signs symbolizing the nature of waste.

Biohazards are substances that threaten all living things on earth. The biohazard symbol presented in Figure 1 was remarked as an important public sign, signaling the harms and hazards of entering the specified zone or room [18]. Along with the biohazard sign, the room door must have a label saying “AUTHORISED PERSONNEL ONLY.” The temporary storage room must always be locked and away from the general public's reach. The waste is then collected by the vehicles daily. A ramp must be present for easy transportation. The waste collected is then taken for treatment. The loading of wastes should not be done manually. It is very vital to properly close or tie the bag or the container to avoid any spillage and harm to the handlers, the public, and the environment. The transport vehicle or trolley must be properly covered, and the route used must be the one with less traffic flow [19].

BMW handling staff should be provided with personal protective equipment (PPE), gloves, masks, and boots. BMW retrievers must be provided with rubber gloves that should be bright yellow. After usage, the importance of disinfecting or washing the gloves twice should be highlighted. The staff working in or near the incinerator chamber must be provided with a non-inflammable kit. This kit consists of a gas mask that should cover the nose and mouth of the staff member. The boots should cover the leg up to the ankle to protect from splashes and must be anti-skid [16]. According to the revised BMW management rules, 2016, it is mandatory to provide proper training to healthcare facility staff members on handling BMW. The training should be mandatorily conducted annually. Along with the management step of the color coding for segregation, it is also important for the staff to be trained in record keeping. This practice of record-keeping helps track the total amount of waste generated and the problems that occurred during the management process, thus helping improve segregation, treatment, and disposal [20].

Color coding for segregation of BMW

Color coding is the first step of BMW management. Different wastes are classified into different types, and therefore, they must be handled and disposed of according to their classification. The bins used for waste disposal in all healthcare facilities worldwide are always color-coded. Based on the rule of universality, bins are assigned a specific color, according to which the waste is segregated. This step helps avoid the chaos that occurs when all types of waste are jumbled, which can lead to improper handling and disposal and further result in the contraction of several diseases [21]. The different kinds of categories of waste include sharp waste such as scalpels, blades, needles, and objects that can cause a puncture wound, anatomical waste, recyclable contaminated waste, chemicals, laboratory waste such as specimens, blood bags, vaccines, and medicines that are discarded. All the above-mentioned wastes are segregated in different colored bins and sent for treatment [22]. Yellow bins collect anatomical waste, infectious waste, chemical waste, laboratory waste, and pharmaceutical waste, covering almost all types of BMW. Different bins and various types of sterilization methods are used depending on how hazardous the waste is. The best tools for sterilization are autoclaves. Red bins collect recyclable contaminated wastes, and non-chlorinated plastic bags are used for BMW collection. Blue containers collect hospital glassware waste such as vials and ampoules. White bins are translucent where discarded and contaminated sharps are disposed of. Sharp wastes must always be disposed of in puncture-proof containers to avoid accidents leading to handlers contracting diseases [23,24]. 

**Figure 3:**
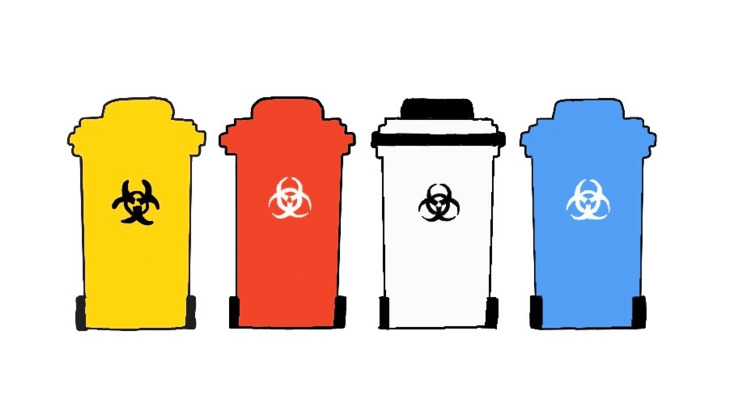
Different colored bins for the segregation of different type of waste [21].

Figure 3 illustrates the different colored bins used for the segregation of BMW.

BMW management refers to completely removing all the hazardous and infectious waste generated from hospital settings. The importance of waste treatment is to remove all the pathogenic organisms by decontaminating the waste generated. This helps in the prevention of many severe health-related issues that can be caused because of the infective waste. It is a method used to prevent all environmental hazards [25].

Methods for the treatment of BMW

There are many methods that are used for the treatment of BMW. One of the most economical ways of waste treatment is incineration, which is just not some simple “burning” but the burning of waste at very high temperatures ranging from 1800℉ to 2000℉ to decrease the total mass of decontaminated waste by converting it into ash and gases, which is then further disposed of in landfills [25,26]. Important instructions associated with the use of incinerators are as follows: chlorinated plastic bags must not be put inside the incinerators as they can produce dioxin [26]. Metals should not be destroyed in an incinerator. The metals present in BMW are made of polyvinyl chloride. When these metals are burned, they produce a huge amount of dioxin. Dioxins are very toxic chlorinated chemical compounds, as dioxins, when released into the environment, can lead to environmental pollution and a higher incidence of cancer and respiratory manifestations [14].

Autoclaving is an alternate method of incineration. The mechanism of this process involved sterilization using steam and moisture. Operating temperatures and time of autoclaving is 121℃ for 20-30 minutes. The steam destroys pathogenic agents present in the waste and also sterilizes the equipment used in the healthcare facility [25]. Autoclaving has no health impacts and is very cost-friendly. It is recommended for the treatment of disposables and sharps, but the anatomical, radioactive, and chemical wastes must not be treated in an autoclave [27]. Chemical methods are the commonest methods that include chemicals such as chlorine, hydrogen peroxide, and Fenton’s reagent. They are used to kill the microorganisms present in the waste and are mainly used for liquid waste, such as blood, urine, and stool. They can also be used to treat solid waste and disinfect the equipment used in hospital settings and surfaces such as floors and walls [28]. Thermal inactivation is a method that uses high temperatures to kill the microorganisms present in the waste and reduce the waste generated in larger volumes. The temperature differs according to the type of pathogen present in the waste. After the treatment is done, the contents are then discarded into sewers [29].

Very serious environmental and health hazards can be triggered if hospital waste is mixed with normal garbage, which can lead to poor health and incurable diseases such as AIDS [30]. The needle sticks can be highly infectious if discarded inappropriately. Injury by these contaminated needles can lead to a high risk of active infection of HBV or HIV [31]. The groups at increased risk of getting infected accidentally are the medical waste handlers and scavengers. Sharps must properly be disposed of in a translucent thin-walled white bin. If sharps are discarded in a thin plastic bag, there is a high chance that the sharps might puncture the bag and injure the waste handler [32]. It can also be the main cause of severe air, water, and land pollution. Air pollutants in BMW can remain in the air as spores. These are known as biological air pollutants. Chemical air pollutants are released because of incinerators and open burning. Another type of threat is water pollutants. BMW containing heavy metals when disposed of in water bodies results in severe water contamination. The landfills where the disposal takes place must be constructed properly, or the waste inside might contaminate the nearby water bodies, thus contaminating the drinking water. Land pollution is caused due to open dumping [33]. BMW must also be kept away from the reach of rodents such as black rats and house mice, which can spread the pathogens to the people living nearby [34].

Many promising steps were taken to minimize the volume of waste discarded from the source, its treatment, and disposal. The 3R system encourages the waste generators to reuse, reduce, and recycle. Everyone must be aware of the 3Rs because this approach can help achieve a better and cleaner environment [35]. Unfortunately, most economically developing countries cannot correctly manage BMW. Very few staff members of healthcare facilities are educated about proper waste management. The waste handlers are also poorly educated about the hazards of waste [36]. Every member helping in the waste management process must be made aware of the dangers of BMW to avoid accidents that harm the environment and living beings [37].

## Conclusions

BMW is generated by healthcare facilities and can be hazardous and infectious. Improper handling can lead to health hazards. Collection, segregation, transportation, treatment, and disposal of BMW are important steps in its management. The color coding of bins, the use of technologies such as incineration and autoclaving, and attention to environmental impacts are also highly crucial. BMW management aims to reduce waste volume and ensure proper disposal. All those involved should strive to make the environment safer.
